# Supplementary material for: Spatial coupling relationship between older adults and elderly care resources in the Yangtze River Delta
Source: PLoS One. 2023 Nov 7;18(11):e0293985. doi: 10.1371/journal.pone.0293985 (PMC10629642; doi:10.1371/journal.pone.0293985)
Supplement: Attachment — Submitted filename: Response to Reviewers.doc [file pone.0293985.s001.doc]

Spatial Coupling Relationship between Older Adults and Elderly Care Resources in the Yangtze River Delta

PONE-D-23-22426

Dear Editors and Reviewers:

Thank you for your careful reading of our manuscript and valuable comments, which helps us significantly improve the revised version of the paper. We believe that we have carefully addressed these comments in the revised version. The details of our responses are shown below, and the texts of the revised manuscript based on the comments are remarked and tracked to facilitate referencing.

Looking forward to hearing from you.

With Best Regards.

#  Response to Reviewer #1

This study explored the spatial coupling relationship between the older adults and elderly care resources in the Yangtze River Delta. I think this manuscript needs substantial revisions before it is considered for publication in PLOS ONE. Here are my comments:

# Comment 1.1

The language of this paper needs to be further polished and improved.

Response 1.1

Thank you for your comment. We have carefully polished the whole paper as you suggested and believe that the revised version is properly written now. First, we change some words in UK English format to their US spelling, such as “ageing” to “aging”, “analyse” to “analyze”, “normalise” to “normalize”, “utilise” to “utilize”. Second, we double check the tense of every sentence. Third, we consider both the positive and negative connotations of words used. For example, terms such as “elderly population” and “elderly people” can sometimes carry negative connotations, so we consistently refer to “elderly population” “elderly people” and “older adult population” as “older adults”. Finally, we make our statements more accurate to avoid confusing readers. For example, we replace the term “elderly care service resources” with “elderly care resources”, “living service resources” with “life service resources”, and “endowment insurance” with “pension insurance”, “aged 65 or over”, “aged 65 or older”, and “aged 65 or above” with “aged 65 and above”. (Details of revisions are remarked in the “Revised Manuscript with tracked change.docx”)

# Comment 1.2

In the section of Introduction, the current situation of population ageing in the Yangtze River Delta should be further illustrated in detail.

Response 1.2

We have added a few sentences to detailedly illustrate the current situation of population aging in the Yangtze River Delta.

“According to the Seventh National Population Census, in 2020, the Yangtze River Delta had a permanent resident population of 230 million, including 35.5 million older adults aged 65 and above, accounting for approximately 15.1% of the total population. The degree of population aging in the Yangtze River Delta is significantly higher than that in the whole country.” (Line 63-66 in the “Revised Manuscript with tracked change.docx”)

**Comment 1.3**

The structure of the paper needs to be improved, especially the Literature Review part. For example, the authors should list the key ideas in the section of literature review. In addition, the logic of some contents in the section of literature review is confusing, such as the second and the third paragraph of the literature review.

**Response 1.3**

Thank you for your comment. We agree that we should list the key ideas in the “Literature Review” and further improve its logic, so we restructure the “Literature Review” by polishing statements and changing the sequence of the existing literature to highlight our key ideas. The logic of the “Literature Review” in the revised version is as follows.

We introduce the existing research of elderly care resources abroad in the first paragraph and display the domestic studies and our key points in paragraphs 2 to 10. In the second paragraph, we elaborate the **conception** of elderly care resources proposed in the existing literature, articulate our definition in broad and narrow perspectives, and further claim that our research focus is material and tangible resources that are accessible to older adults needed for the social elderly care. In the third paragraph, we divide the elderly care resources into **four aspects** based on our research scope and some classifications from other scholars. In the fourth paragraph, we state the **unbalance and insufficiency** of social elderly care resources in China, indicating the necessity to study the relationship between older adults and elderly care resources and their reasonable allocation. In the fifth paragraph, we claim that the **supply-and-demand** relationship of elderly care resources is an important index to reflect their allocation, and coupling theory is one of tools to study the relationship between supply and demand, which is the methodology adopted in our study. In paragraphs 6-8, we summarize the research **deficiencies** concerning the research **content, method, and scope** in the existing literature, and further propose our research **contributions and interests** in paragraphs 9 and 10. Thus, our **key points** are shown in three aspects: first, we propose our definition of elderly care resources in the second paragraph; second, we present its division in the third paragraph; third, we elaborate more on the contributions, significance, methods, motivation and focus in paragraphs 9 and 10. (Here is the overall explanation of the segmented content, and detailed adjustments and modifications can be found in Line 73-208 in the “Revised Manuscript with tracked change.docx”)

**Comment 1.4**

It would be better to include a map to show the research area.
**Response 1.4**

We have added “Fig 1” in Line 62, 71 and 198 and adjusted the order of the remaining figures and their descriptions in the paper accordingly.

**Comment 1.5**

The authors should check their manuscript more carefully before submitting the manuscript. There are some obvious flaws in this manuscript, such as Line 190 and Line 248.

**Response 1.5**

Thank you for your reminder. We have corrected these errors and performed an extra check as suggested by the referee. (Line 228 and 306 in the “Revised Manuscript with tracked change.docx”)

**Comment 1.6**

Table 1: It would be better if the authors define the scope of different facility resources in more details. For example, what types of infrastructure facilities do the “elderly care facilities” contain? In addition, can the indicator of “number of medical beds per 10,000 older adults” fully represent the medical and health resources needed by older adults?

**Response 1.6**

(1) Thank you for the reviewer's valuable suggestions. We have added the definition of the scope of elderly care facilities in life service resources which is the first type of elderly care resources and provided examples in the paper. The details are as follows：

It should be noted that, according to the reference [1] and the principles of data accessibility, the elderly care facilities in this study, which are categorized under life service resources, primarily refer to the resources of social elderly care institutions that provide various services and support for the older adults. The elderly care facilities contain elderly apartments, elderly daycare institutions, nursing stations, nursing homes, medical institutions. Based on the Earth Big Data, the data of elderly care facilities can be extracted using Python. (Line 260-265 in the “Revised Manuscript with tracked change.docx”)

1. *Huang X, Gong P, White M. Study on spatial distribution equilibrium of elderly care facilities in downtown Shanghai. Int J Environ Res Public Health, 2022, 19(13): 7929.*

(2) The feedback from the reviewers is very reasonable.

**Firstly,** many studies utilize variables such as the number of institutions, the number of beds, and the number of technicians to represent the medical and health resources [Error: Reference source not found-Error: Reference source not found]. The common feature among these studies is that most of them have utilized the indicator of "number of medical beds per 10,000 older adults" to reflect the accessibility of medical and health resources. To a certain extent, this indicates that " the number of medical beds per 10,000 older adults" can serve as a representative measure of medical and health resources.

Considering that there are similar issues with the selection of other indicators, we have added literature basis and principles for indicator selection in the paper as follows:

“Based on the principles of scientificity, feasibility, availability, and comparability [1], we select the following evaluation indicators[Error: Reference source not found-5]: the number of elderly care facilities per 10,000 older adults (, the area of park green spaces per 10,000 older adults (, the number of urban basic old-age insurance participants per 10,000 older adults (, and the number of medical beds per 10,000 older adults (, corresponding to the four second-level indicators, respectively.……” (Line 251-259 in the “Revised Manuscript with tracked change.docx”)

**Secondly,** we consider the accessibility and availability of data, as not all resources have comprehensive data for multiple variables and considering the comparability issues among various types of resources, each type of elderly care resource is measured using a single variable. Therefore, this study adopts the indicator “the number of medical beds per 10,000 older adults" to represent the medical and health resources required by older adults.

**Thirdly,** we would like to express our gratitude for the reviewer's valuable suggestions. Indeed, this paper has certain limitations in adequately representing different types of elderly care resources due to the difficulty to obtain data for some variables. Hence, in the "Conclusions and Discussion" section, we have added the following statement:

"Furthermore, the challenge of data accessibility and comparability of four types of elderly care resources led us to choose only one indicator to represent each resource. There may be a potential limitation in adequately characterizing four kinds of elderly care resources, so future research needs to consider increasing types of mental elderly care resources to improve the classification of elderly care resources and consider more indicators for each elderly care resource，so as to comprehend the status of various elderly care resources as comprehensively and deeply as possible. (Line 637-645 in the “Revised Manuscript with tracked change.docx”)

2. *Huang X, Gong P, White M. Study on spatial distribution equilibrium of elderly care facilities in downtown Shanghai. Int J Environ Res Public Health, 2022, 19(13): 7929.*
3. *Zhang Y, Wang Q, Jiang T, Wang J. Equity and efficiency of primary health care resource allocation in mainland China. Int J Equity Health. 2018 Sep 12;17(1):140. doi: 10.1186/s12939-018-0851-8. PMID: 30208890; PMCID: PMC6134520.*
4. *Prinja S, Gupta A, Verma R, Bahuguna P, Kumar D, Kaur M, Kumar R. Cost of delivering health care services in public sector primary and community health centres in North India[J]. PloS one, 2016, 11(8): e0160986.*
5. *Lin L, Wu F, Chen W, Zhu C, Huang T. Research on urban medical and health services efficiency and its spatial correlation in China: based on panel data of 13 cities in Jiangsu Province[C]//Healthcare. MDPI, 2021, 9(9): 1167.*
6. *Shao Q, Yuan J, Lin J, Huang W, Ma J, & Ding H. A SBM-DEA based performance evaluation and optimization for social organizations participating in community and home-based elderly care services[J]. PloS one, 2021, 16(3): e0248474.*

**Comment 1.7**

In the manuscript, the terms of “elderly care resources”, “elderly care service resources” and “elderly care facilities resources” are very confusing to the reader.

**Response 1.7**

Thanks for your advice. We are sorry that it is actually confusing to the reader. Therefore, we proofread the whole paper and unify these terms as you suggested. We replace the “elderly care service resources” as “elderly care resources” (in the whole paper of “Revised Manuscript with tracked change.docx”). In our study, elderly care resources refer to material and tangible resources that are accessible to older adults.

To avoid confusing readers, we add “The sequence of elderly care resources has four indicators: life service resources, ecological environment resources, social security resources, and medical and health resources (see Table 1)” (Line 250-252 in the “Revised Manuscript with tracked change.docx”). As mentioned above (Response1.6), the elderly care facilities in this study, which are categorized under life service resources, primarily refer to the resources of social elderly care institutions that provide various services and support for the older adults, such as elderly apartments, elderly daycare institutions, nursing stations, nursing homes, medical institutions (Line 260-265 in the “Revised Manuscript with tracked change.docx”). To avoid confusing readers, we change the second-level indicator “Elderly care facility resources” in Table 1 to life service resources (the second-level indicator, the first type of elderly care resources) but retain the third-level indicator “the number of elderly care facilities per 10,000 older adults” to measure the abundance of life service resources. (Page 13 Table 1 in the “Revised Manuscript with tracked change.docx”)

**Comment 1.8**

Line 419: What is “life service resources”? There is a mismatch between the text and Table 1 in terms of the names of indicators.

**Response 1.8**

(1) Life service resources are various services provided to meet the daily needs of older adults [Error: Reference source not found], such as institution and community elderly care facilities. Furthermore, we have added the definition of four types of elderly care resources in the “**Literature review**”: life service resources, ecological environment resources, social security resources, and medical and health resources. (Line 113-120 in the “Revised Manuscript with tracked change.docx”)

(2) Thanks for the advice. Yes, we are very sorry that there is a mismatch between the names of indicators. We have corrected the text above Table 1 and make all indicators more accurate.

“The sequence of elderly care resources has four indicators: life service resources, ecological environment resources, social security resources, and medical and health resources (see Table 1).” (Line 250-252 in the “Revised Manuscript with tracked change.docx”)

We change “Elderly care facility resources” to “life service resources” (the second-level indicator, the first type of elderly care resources), meanwhile, change “Park green space resources” to “Elderly care facility resources” (the second-level indicator, the second type of elderly care resources). (Table 1, Page 13 in the “Revised Manuscript with tracked change.docx”)

**Comment 1.9**

In the section of conclusion, the authors should list more targeted policy implications based on the analysis results. In addition, some implications seem to be unrealistic, such as Line 534: it is necessary to reasonably guide older adults to move to areas with rich elderly care resources and low aging levels.

**Response 1.9**

Thank you for the reviewer's advice. Both Strategy 2 and Strategy 3 in the “**Conclusions and discussion”** are targeted suggestions based on the analysis results in the paper, but there are still problems such as insufficient targeting and unrealistic solutions just as suggested by the reviewer.

With the implementation of the Yangtze River Delta integration strategy, the integration of transportation in the Yangtze River Delta (through Shanghai and Suzhou subways) and the integration of medical care in the Yangtze River Delta (through the opening of long-distance medical insurance) have all provided feasibility for the integration of elderly care in the Yangtze River Delta. Therefore, this strategy has certain feasibility. However, the reviewers’ suggestions are reasonable. This countermeasure is only useful for the older adults in large cities (such as Shanghai) to go to nearby places (such as Suzhou) for elderly care, but not applicable to the older adults in Nantong. In addition, it is not realistic to let the older adults in Shanghai go to Hefei, Huangshan and other places for elderly care, so the relevant contents in the article have been deleted: Line 626 deleted "North Anhui"; Line 628-630 deleted "tourist cities with rich ecological environment resources and a sparse elderly population (such as Huangshan);" and “Hefei with a relatively low density of older people and”; Line 631 deleted " and Nantong ".

The last suggestion should emphasize that elderly people in Shanghai should go to the surrounding areas of Shanghai (such as Suzhou) for elderly care, thereby excluding other regions such as Nantong and increasing the feasibility of implementing this strategy. Therefore, it is revised to "Finally, it is necessary to reasonably guide older adults **in Shanghai** to move to **nearby** areas with rich elderly care resources and low aging levels for elderly care.” (Line 619-620 in the “Revised Manuscript with tracked change.docx”)

**Comment 1.10**

The authors need to elaborate more on the contribution of this paper to our knowledge. Given the limited available spaces for older adults’ daily activities, does it make sense to carry out analysis at such a large spatial scale?

**Response 1.10**

(1) We have added the contribution of this paper in “**Literature review**”: “In terms of content, this paper researches the coupling and coordinated development of elderly care resources as a whole, integrate all elderly care resources, and explore the path to optimize the spatial allocation of elderly care resources. It can enrich the theoretical connotations of balanced development, sustainable development, resource sharing, and regional integration of elderly care resources, laying a theoretical foundation for the development of gerontology. In terms of research theory and methods, this study integrates interdisciplinary theories and methods such as geography, statistics, and management, which is conducive to eliminating disciplinary barriers and promoting the development of interdisciplinary theories and methods. In terms of research scope and countermeasures, this study utilizes the integrated national strategy of the Yangtze River Delta, selects national key regions such as the Yangtze River Delta as the research object, and proposes that optimizing elderly care resources in the Yangtze River Delta based on the integration of the Yangtze River Delta is conducive to breaking through local protectionism and administrative barriers, and transitioning from "regionalization" and "fragmentation" governance to "integration" and "integrity" elderly care social governance models”. (Line 186-197 in the “Revised Manuscript with tracked change.docx”)

(2) Although the available space for daily activities of the elderly is limited, it is of great theoretical and practical significance to analyze the coupling development relationship between elderly care resources and older adults at the spatial scale of the Yangtze River Delta as population mobility, especially the increasing frequency of elderly population mobility, and the integration of the Yangtze River Delta has become a national strategy. Reasonably improving the spatial allocation of elderly care resources is a practical and feasible solution to the practical problems of elderly care in China. In the context of the integration of the Yangtze River Delta, integrating elderly care resources within the Yangtze River Delta, strengthening cooperation in elderly care services and older adults in Shanghai to provide elderly care in nearby areas, are conducive to promoting the integrated development of elderly care in the Yangtze River Delta, overcoming the bottleneck of current Shanghai elderly care resource restrictions, alleviating the pressure on elderly care and resource supply burden in Shanghai, and reducing its adverse impact on the economy and society. At the same time, the abundant labor resources in other cities of the Yangtze River Delta except Shanghai can provide elderly care service talents for Shanghai's elderly care resources. The integration of elderly care resources and cooperation in elderly care services in the Yangtze River Delta will promote the development of the elderly care industry and related industries, which is conducive to fully leveraging the industrial linkage effect, thus promoting the economic and social reform and development of Shanghai and the entire Yangtze River Delta region.

# Response to Reviewer #2

I would like to pay my thanks to you for entrusting me with this opportunity to review an article for your journal. After reading this article critically, I think this article is very clear and well written. The Yangtze River Delta is an important economic zone in China and also one of the regions with severe population aging issues. The relationship between elderly care resources and older adults is crucial for China's strategy to address population aging. Therefore, the study is pretty important in the context of China. The authors put their best into conducting this research. The abstract, introduction, literature and results are well written and explained. Though, I have a few suggestions which are expected to be considered in the revision of the manuscript and future research.

**Comment 2.1**

(1) Third-level indicators of the coupling system need to be chosen more carefully and need to be elaborated.

**Response 2.1**

Thank you for your suggestion. With reference to the existing research, we have added several sentences to elaborate the third-level indicators as follows:

“Based on the principles of scientificity, feasibility, availability, and comparability [36], we select the following evaluation indicators[37-43]: the number of elderly care facilities per 10,000 older adults ($Y_{1}$), the area of park green spaces per 10,000 older adults ($Y_{2}$), the number of urban basic old-age insurance participants per 10,000 older adults ($Y_{3}$), and the number of medical beds per 10,000 older adults ($Y_{4}$) , corresponding to the four second-level indicators, respectively. The more elderly care facility institutions, the more abundant life service resources are available to the older adults; the more park green spaces every older adult owns, the more friendly communities will be developed; the more participants, the more social security resources are supplied; the more medical beds, the more elderly inpatients can be adopted [31]”. (Line 252-259 in the “Revised Manuscript with tracked change.docx”)

**Comment 2.2**

In the "Literature Review", it was mentioned that the national strategy of "Yangtze River Delta Integration" is particularly important for promoting the integration of elderly care resources. However, the importance of "Yangtze River Delta Integration" in promoting the integration of elderly care resources and how to use the Yangtze River Delta Integration Strategy to promote the integration of elderly care resources have not been clearly reflected in the countermeasures. It is necessary to highlight or supplement the suggestions in the countermeasures.

**Response 2.2**

Thank you very much for your valuable suggestions. In the second countermeasure, we add relevant suggestions for the Yangtze River Delta integration strategy to promote the integration of elderly care resources.

“Second, based on the integration strategy of the Yangtze River Delta, strengthen cooperation in elderly care services in the Yangtze River Delta, integrate elderly care resources in the regions within the Yangtze River Delta, adjust the spatial allocation of elderly care resources, and promote the integrated development of elderly care in the Yangtze River Delta.” (Line 599-602 in the “Revised Manuscript with tracked change.docx”)

“For the Yangtze River Delta region, measures should be taken to ensure that Shanghai plays a leading role in high-tech and high medical level, accelerate the implementation of the Yangtze River Delta integration strategy, strengthen regional connections, and promote the integrated development of smart elderly care in the Yangtze River Delta. Accelerate the implementation of the Yangtze River Delta integration strategy, promote the integration of transportation in the Yangtze River Delta (such as the passage of Shanghai and Suzhou subways), and the integration of medical care in the Yangtze River Delta (such as the opening of remote medical insurance), providing feasibility for the integration of elderly care in the Yangtze River Delta.” (Line 611-617 in the “Revised Manuscript with tracked change.docx”)

**Comment 2.3**

At present, there is a lack of literature in the reference list that has been submitted to the journal. Therefore, it is recommended to appropriately add references to the relevant topics that have been published in the PLOS ONE.

**Response 2.3**

Thank you for your kind reminder. We have added 5 references published in the PLOS ONE and 5 references published in other journals to support our theory and results in “**References**”. The details are as follows: (Line 649-763 in the “Revised Manuscript with tracked change.docx”).

(1) 5 references published in the PLOS ONE:
1. Sasaki I., Kondo K., Kondo N., Aida J., Ichikawa H., Kusumi, et al. Are pension types associated with happiness in Japanese older people?: JAGES cross-sectional study. PLoS One, 2018, 13(5): e0197423.
2. Salinas-Rodríguez A., Torres-Pereda M. D. P., Manrique-Espinoza B., Moreno-Tamayo K., & Téllez-Rojo Solís. Impact of the non-contributory social pension program 70 y más on older adults’ mental well-being. PloS one, 2014, 9(11): e113085.
3. Prinja S, Gupta A, Verma R, Bahuguna P, Kumar D, Kaur M, Kumar R. Cost of delivering health care services in public sector primary and community health centres in North India. PloS one, 2016, 11(8): e0160986.
4. Shao Q, Yuan J, Lin J, Huang W, Ma J, & Ding H. A SBM-DEA based performance evaluation and optimization for social organizations participating in community and home-based elderly care services. PloS one, 2021, 16(3): e0248474.
5. Zhang X, Zhao L, Cui Z, Wang Y. Study on Equity and Efficiency of Health Resources and Services Based on Key Indicators in China. PLoS One. 2015 Dec 17;10(12):e0144809. doi: 10.1371/journal.pone.0144809. PMID: 26679187; PMCID: PMC4683010.

(2)5 references published in other journals:

1. Wu F, Chen W, Lin L, Ren X, Qu Y. The Balanced allocation of medical and health resources in urban areas of China from the perspective of sustainable development: a case study of Nanjing. Sustainability, 2022, 14(11): 6707.
2. Zhang Y, Wang Q, Jiang T, Wang J. Equity and efficiency of primary health care resource allocation in mainland China. Int J Equity Health. 2018 Sep 12;17(1):140. doi: 10.1186/s12939-018-0851-8. PMID: 30208890; PMCID: PMC6134520.
3. Du X J, Lin H H, Hsu I C, et al. River green land and its influence on urban economy, leisure development, ecological protection, and the well-being of the elderly. Water, 2023, 15(7): 1350.
4. Lin L, Wu F, Chen W, Zhu C, Huang T. Research on urban medical and health services efficiency and its spatial correlation in China: based on panel data of 13 cities in Jiangsu Province[C]//Healthcare. MDPI, 2021, 9(9): 1167.
5. Yi M, Peng J, Zhang L, Huang W, Ma J, & Ding H. Is the allocation of medical and health resources effective? Characteristic facts from regional heterogeneity in China. International Journal for Equity in Health, 2020, 19(1): 1-21.
